# Functional invadopodia formed in glioblastoma stem cells are important regulators of tumor angiogenesis

**DOI:** 10.18632/oncotarget.25045

**Published:** 2018-04-17

**Authors:** Christos Petropoulos, Pierre-Olivier Guichet, Konstantin Masliantsev, Michel Wager, Lucie Karayan-Tapon

**Affiliations:** ^1^ INSERM U1084, Laboratoire de Neurosciences Expérimentales et Clinique, Poitiers, F-86073, France; ^2^ Université de Poitiers, Poitiers, F-86073, France; ^3^ CHU de Poitiers, Laboratoire de Cancérologie Biologique, Poitiers, F-86022, France; ^4^ CHU de Poitiers, Service de Neurochirurgie, Poitiers, F-86021, France

**Keywords:** glioblastoma, invadopodia, angiogenesis, CD44, LIMKs

## Abstract

Glioblastoma (GBM) represents the most common and lethal brain tumor. High vascularization, necrosis and invasiveness are hallmarks of GBM aggressiveness with recent data suggesting the important role of glioblastoma stem cells (GSCs) in these processes. It is now well established that cancer cells employ specialized structures termed invadosomes to potentiate invasion. However, the role of these structures in GBM dissemination remains poorly investigated. In this study, we showed that GBM-isolated GSCs form invadopodia-like protrusions endowed with degradative action. Interestingly, their formation depends on extracellular matrix (ECM) sensing via the CD44 receptor. We also found that GSCs invasive migration occurring during tubes assembly is promoted through invadopodia-mediated-ECM remodeling and LIM kinases signaling. Moreover, our study demonstrates that GSCs are highly adaptable cells that are able not only to restore damaged endothelial-derived tubes but also to generate in cooperation with normal endothelial cells (ECs) intact vascular channels. Taken together, our data provide new insights in GBM microvasculature and suggest that GSCs targeting in combination with anti-VEGF therapy may block tumor progression.

## INTRODUCTION

Glioblastomas (GBMs) are the most aggressive and fatal primary tumors. Despite the progress made in therapeutic modalities, GBM treatment remains insufficient due to rapid tumor recurrence [1]. GBM cells escaping from the primary tumor have the capacity to rapidly invade into the normal brain parenchyma and promote the formation of relapsed tumors. There is now compelling evidence that GBM invasiveness and radio/chemo-resistance is strongly correlated with a subpopulation of self-renewing, multi-potent and tumor-initiating cells termed glioblastoma stem cells (GSCs) [2, 3]. GSCs share a variety of stem-cell markers such as CD133, Nestin and CD44 with normal neural stem cells [4, 5]. Adult neural stem cells inhabit within protective microenvironments or niches that maintain stem cells in a quiescent and undifferentiated state [6, 7]. Like normal stem cells, GSCs are found to reside in vascular niches where endothelial-derived factors and osteopontin ligands maintain GSCs stemness [8, 9].

Several studies have reported that GBM cells generate invasion paths into the nerve tissue via the secretion of proteolytic enzymes such as matrix metalloproteinases (MMPs) into the extracellular space [10–12]. It is now well established that MMPs secretion is spatially restricted to specialized structures formed on the ventral cellular side known as invadosomes. They represent dot-like structures of filamentous actin endowed with ECM degrading activity [13–15]. Typically, invadosomes are referred to as podosomes when formed in normal cells (monocytic cells, osteoclasts and endothelial cells) and invadopodia when found in cancer cells [16–18]. Even though invadosomes have been directly connected with invasive processes, their role in GBM invasion is poorly explored.

GBMs are high vascular tumors and until 2010, it has been reported, that tumor vasculature arises by sprouting of pre-existing brain capillaries and that GSCs trigger vessel neo-formation by secreting angiogenic factors [19]. However, new data revealed that GSCs are capable of differentiation into endothelial cells (ECs) in order to support tumor vascularization [20–22]. Since endothelial-based niches are responsible for GSCs maintenance and GBM vascularization, endothelium–targeting has been a major focus of research, drug discovery, and clinical treatment [23]. Even though angiogenesis inhibitors targeting VEGF-signaling had antitumor effects, these therapies concluded to tumor adaptation and recurrence [24].

As GSCs are considered to be key players for tumor propagation, we assessed whether invadopodia formation in GSCs contribute to their invasive character. Herein, we confirmed that GSCs form invadopodia-like protrusions in certain regions where ECM degradation also occurs. Importantly, invadopodia assembly in GSCs depends on ECM signaling mediated by CD44 receptors. *In vitro* angiogenesis assays were then applied to examine GSCs capacity to form capillary-like networks. Gelled substrates triggered GSCs invasive migration that resulted in tubes formation reminiscent of normal endothelium. Additional *in vitro* assays revealed that GSCs are highly adaptable cells able not only to restore damaged endothelial-derived tubes but also to promote angiogenesis in cooperation with normal endothelial cells. Moreover, we demonstrated for the first time that fully functional invadopodia formed in GSCs permitted gelled matrix remodeling and consequently tubes formation. We also showed that during tubes assembly LIMKs signaling is upregulated and highly required for GSCs invasive migration. Taken together, these findings indicate that GSCs due to their cellular plasticity exert important roles during tumor vascularization.

## RESULTS

### GSCs upon adhesion form invadopodia

**Figure 1:**
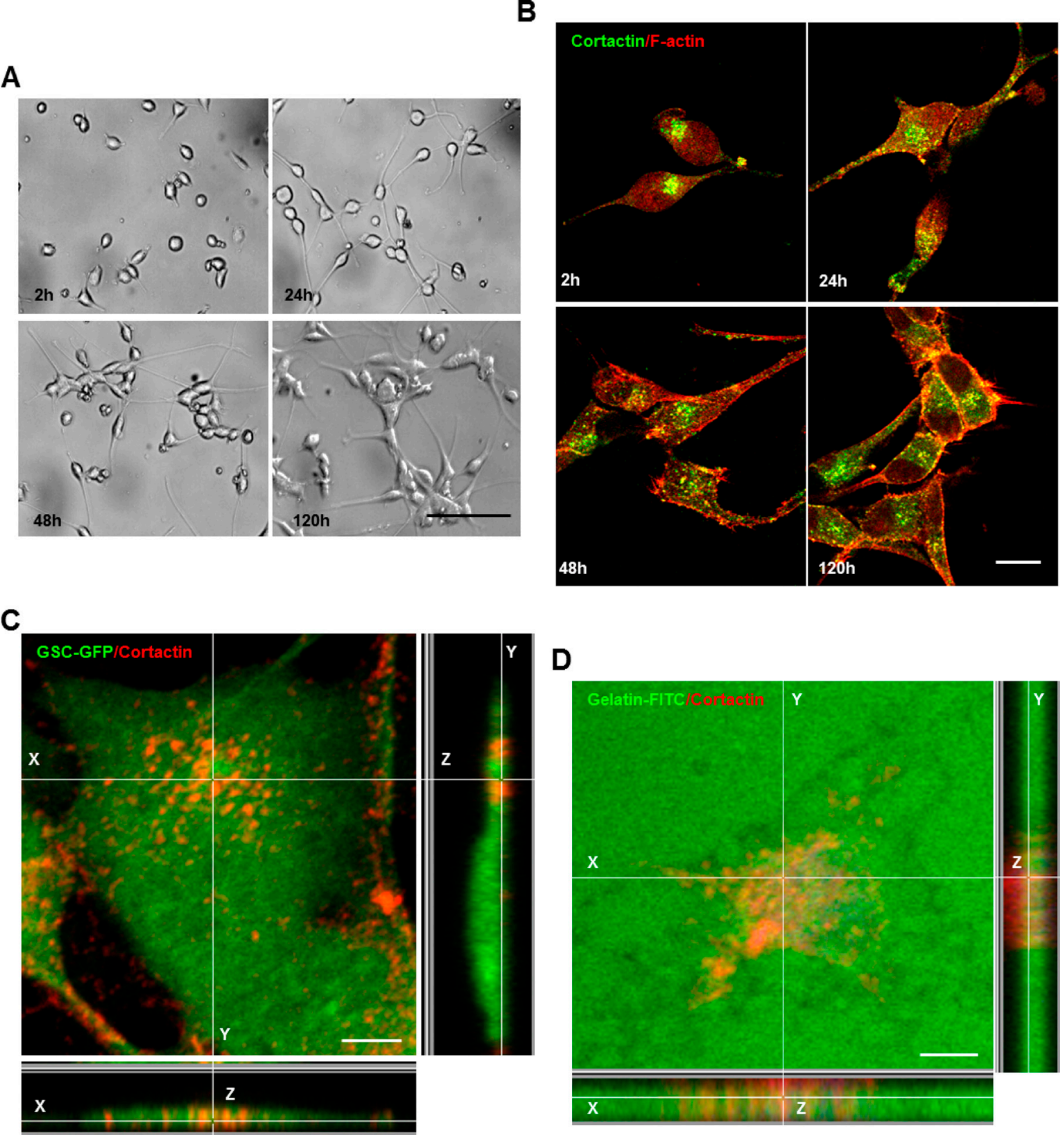
GSCs upon adhesion form invadopodia (**A**) GSCs were seeded on matrigel-coated coverslips for different time periods 2, 24, 48 and 120 h. (**B**) GSCs were fixed and stained with anti-cortactin antibody (green) and rhodamine phalloidin (red). GSCs adhesion on matrigel was accompanied by invadopodia formation. (**C**) GFP-expressing GSCs were stained with cortactin antibody (red) and analyzed with confocal microscopy. Z-sectioning showed cortactin staining at columnar structures rising perpendicular to the substratum. (**D**) GSCs were plated on fluorescent (green) gelatin-coated coverslips for 16 h before fixation and staining with cortactin (red). Confocal imaging demonstrated that matrix degradation occurred in regions where cortactin-containing invadopodia protruded into the gelatin layer. Bars: (A) 100 μm; (B) 30 μm; (C) 50 μm; (D) 50 μm.

Even though several studies have highlighted the importance of invadopodia in cancer cell invasion, these structures remain poorly investigated in GBM. Therefore, we assessed invadopodia formation in GSCs (GSC2 line) derived from an adult GBM-patient. Under serum-free culture conditions, GSCs proliferate as non-adherent multicellular spheroids (Supplementary Figure 1A). To determine whether GSCs form invadopodia, GSC-spheres were dissociated and isolated cells were cultured on matrigel for different time periods (2, 24, 48 and 120 h). Matrigel substrates not only triggered GSCs adhesion, but also promoted filopodium-like protrusions formation and cell clustering (Figure 1A). Double immunostaining with cortactin (core component) and phalloidin (F-actin probe) confirmed GSCs capacity to form invadopodia (Figure 1B). To assess that the cortactin-containing cores represent columnar structures on the ventral cellular side, confocal imaging was performed. Z-sectioning clearly showed that these cortactin-rich puncta represented columnar structures rising perpendicular to the substratum (Figure 1C). This observation was further confirmed by immunofluorescence analysis of GSCs plated on FITC-labeled gelatin-coated coverslips. Indeed, these cortactin-rich structures protruded into the gelatin layer in regions where ECM degradation also occurred (Figure 1D). To determine if adhesion stimulated GSC differentiation we examined Nestin and SOX2 expression in fixed cells plated on matrigel. Even at 120 h post-seeding on matrigel, GSCs retained their stem-cell phenotype (Supplementary Figure 1B). These findings prove clearly that invadopodia formation in GSCs could explain their significant invasive behavior.

### ECM signaling mediated by CD44 controls invadopodia assembly

**Figure 2:**
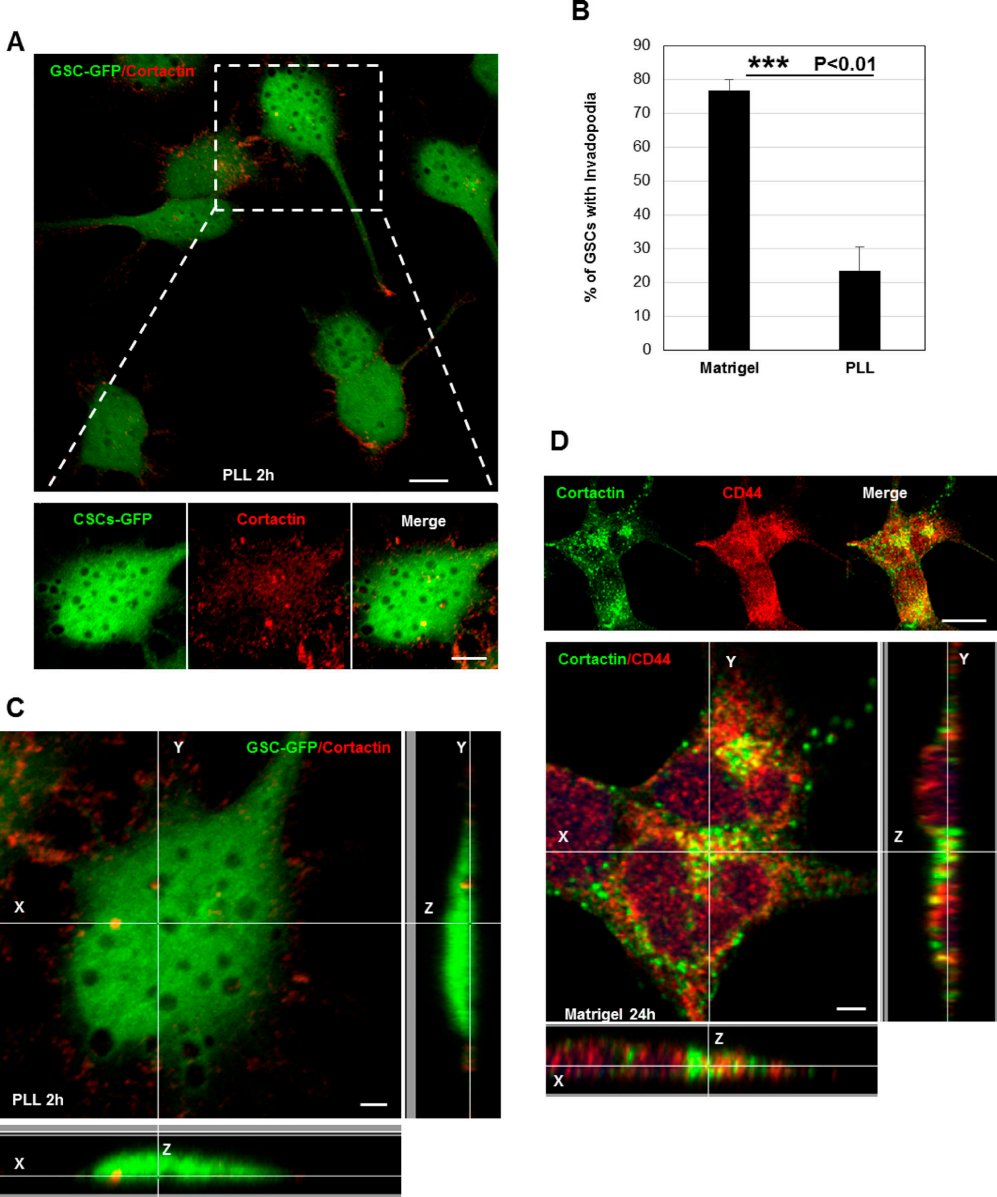
ECM signaling mediated by CD44 controls invadopodia assembly (**A**) GFP-expressing GSCs were plated on PLL-coated coverslips for 2 h and stained for cortactin (red). Extracellular matrix sensing by cell-surface receptors controls invadopodia assembly. (**B**) Quantification of the % of GSCs with invadopodia cultured on PLL- or Matrigel-coated coverslips. In the absence of extracellular proteins (PLL substrates) GSCs were unable to form invadopodia ^***^P < 0.01 (*n =* 3). (**C**) Z-sectioning confirmed the absence of cortactin-positive protrusions on the ventral cellular side of GSC-GFP cells seeded on PLL. (**D**) GSC cells grown on matrigel were stained with cortactin (green) and CD44 (red) antibodies. Immunofluorescence images showed that CD44 colocalized with cortactin at invadopodia sites. Z-stack analysis confirmed CD44 localization to cortactin-containing protrusions at the lateral cellular side. Graph presented as means ± SD. Differences with a probability level *P* < 0.05 were considered significant in one-way ANOVA. Bars: (A) 20 μm; 5 μm; (C) 4 μm; (D) 30 μm.

**Figure 3:**
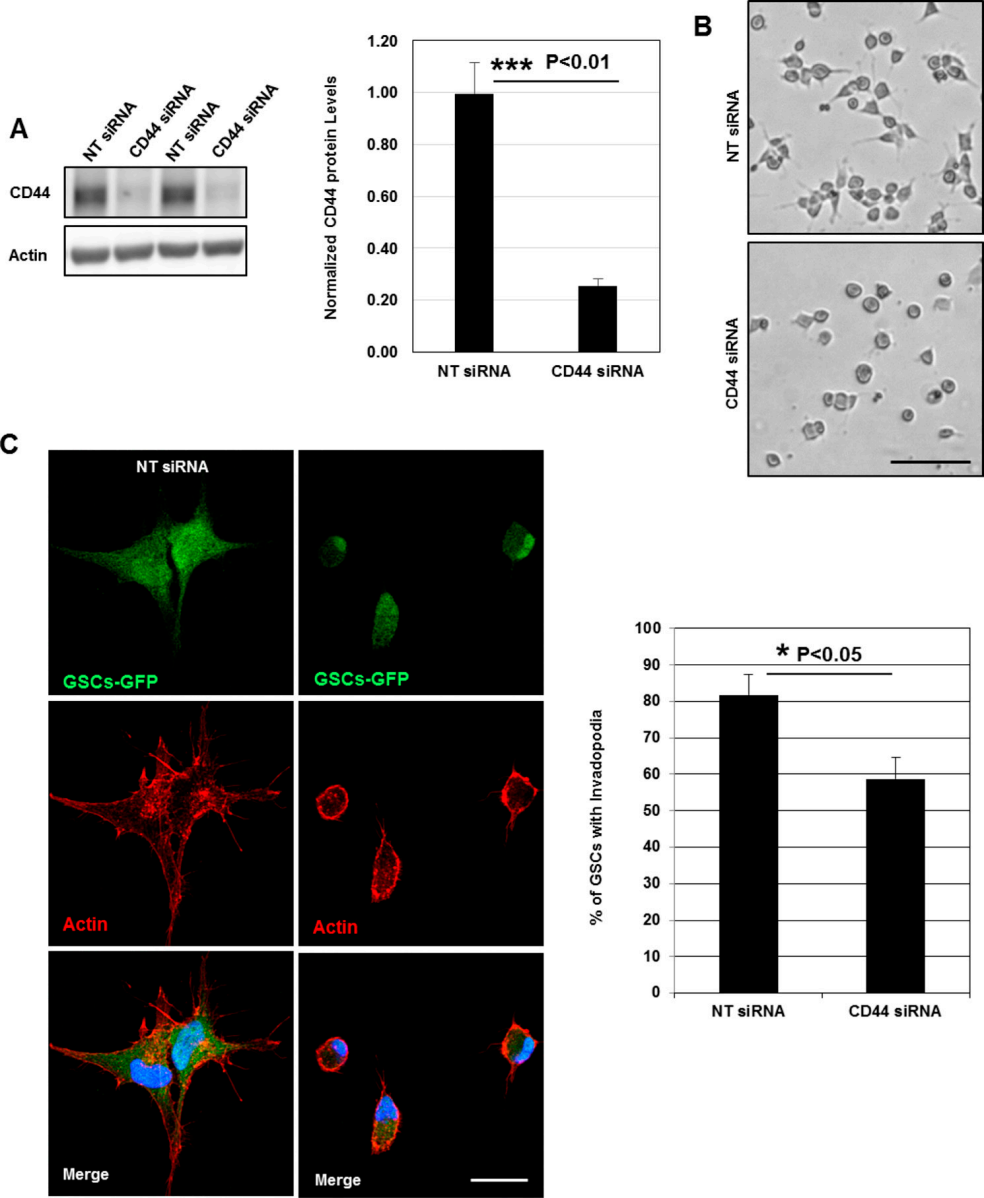
CD44 outside-in signaling controls invadopodia formation (**A**) Cell lysates of GSCs transfected with NT- or CD44- siRNA were analyzed by Western blotting and probed for CD44 and Actin. SiRNA silencing strategy was efficient leading to 75% decrease in CD44 protein levels; ^***^P < 0.01 (*n =* 3). (**B**) CD44-siRNA knockdown affected GSCs spreading capacity on Matrigel-coated substrates. (**C**) NT- or CD44- siRNA transfected GSC-GFP cells were fixed and stained with rhodamine phalloidin and DAPI. CD44 rapid depletion strongly affected cells ability to form invadopodia. CD44 knockdown significantly reduced the % of GSCs with invadopodia; ^*^P < 0.05 (*n =* 3). Graphs presented as means ± SD. Differences with a probability level *P* < 0.05 were considered significant in one-way ANOVA. Bars: (B) 50 μm; (C) 30 μm.

Despite the fact that invadopodia represent specialized cell-matrix contacts, it is unknown whether ECM signals regulate their assembly [17]. To address the role of ECM signaling on invadopodia formation, we plated GSCs on poly-L-lysine (PLL)-coated coverslips. Cell attachment on PLL is independent of surface receptors and occurs via electrostatic interactions. Interestingly, we observed that GSCs (cultured for 2 h) were unable to form invadopodia on PLL as compared to cells seeded on matrigel (Figures 2A, 2B and 1B). Confocal z-sectioning further confirmed the absence of columnar structures suggesting that ECM signaling (outside-in signaling) transmitted via surface receptors control their formation (Figure 2C). Because in GSCs CD44 receptor is highly expressed, we wanted to define its role in invadopodia [4, 5, 9]. Therefore, GSCs were plated on matrigel substrates and we analyzed whether CD44 is a component of the invadopodium structure. Immunofluorescence analysis showed that CD44 colocalized with cortactin at invadopodia sites (Figure 2D). Further z-stack analysis also revealed that CD44 localized to cortactin-containing columnar structures at the lateral cellular side. To determine whether CD44 plays an important role in invadopodia initiation process in GSCs, a silencing strategy targeting CD44 receptor was applied. SiRNA treatment was efficient, resulting in 74% decrease in CD44 protein levels (Figure 3A). Interestingly, we observed that CD44 silencing strongly affected GSCs spreading since CD44-siRNA cells presented a rounded morphology relative to the elongated form exhibited by control-treated cells (NT; non-targeting siRNA) (Figure 3B). Notably, CD44 rapid depletion significantly decreased GSCs capacity to form invadopodia. Moreover, staining with actin revealed that CD44-siRNA GSCs exhibited a reduced invadopodia number compared to NT-treated cells (Figure 3C). Thus, these data suggest that ECM signals transmitted via CD44 promote invadopodia formation.

### The CD44 ligand osteopontin (OPN) controls invadopodia assembly

**Figure 4:**
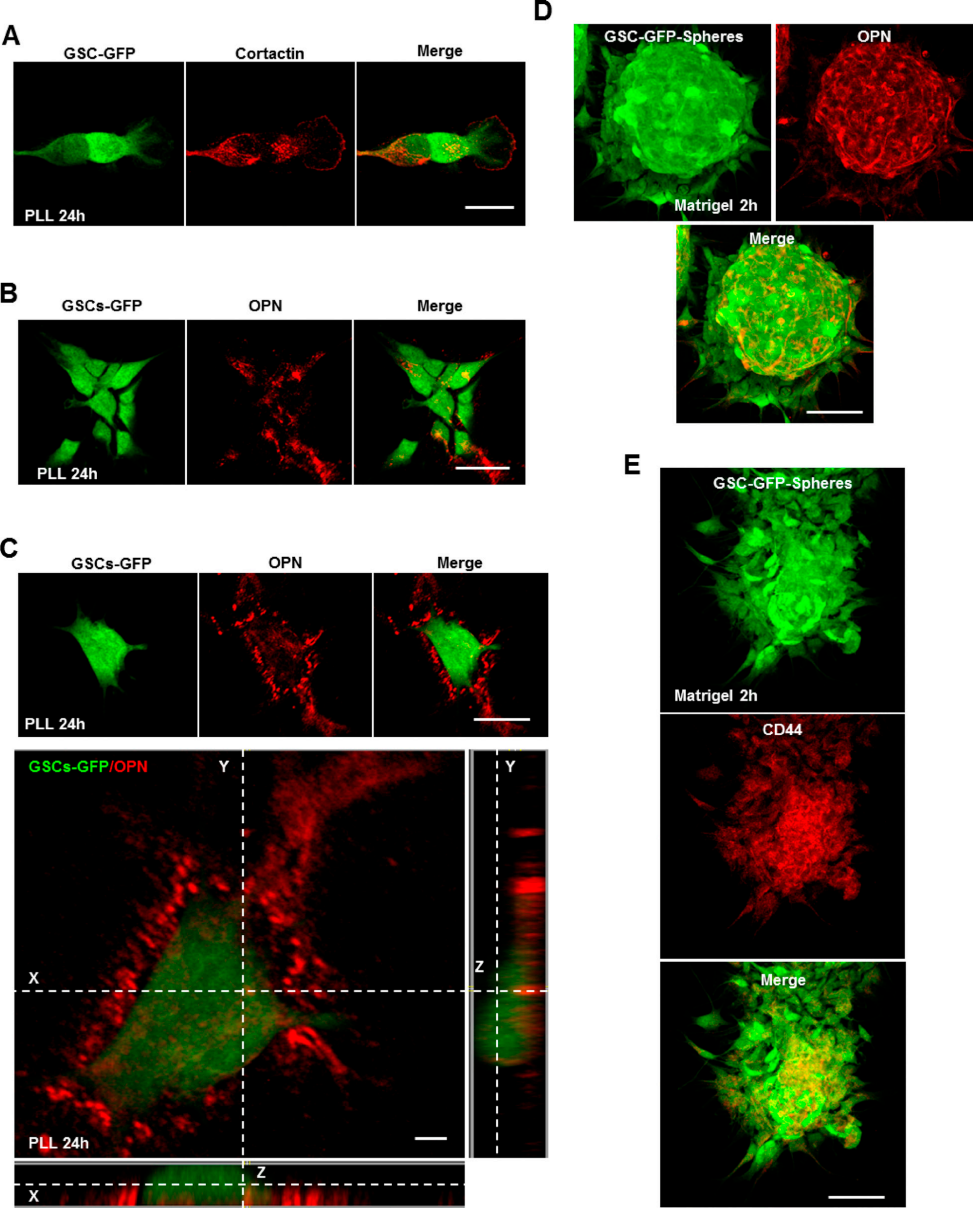
The CD44 ligand OPN controls invadopodia assembly (**A**) GFP-expressing GSCs were seeded on PLL-coated coverslips for 24 h and stained for cortactin (red). GSCs cultured on PLL for 24 h presented numerous invadopodia puncta. (**B**) GSC-GFP cells were plated on PLL for 24 h and then stained for secreted OPN ligands (red). Immunofluorescence images revealed OPN localization (red) to regions where GSC-GFP cells adhered. (**C**) Z-stack analysis confirmed OPN localization to the extracellular space underneath the cells. (**D**) GSC-GFP spheres were cultured on matrigel-coated coverslips for 2 h, fixed and stained with an OPN antibody (red). Immunofluorescence analysis showed OPN enrichment within the multicellular bulk. (**E**) GFP-expressing spheres were plated on matrigel-coated substrates for 2 h and stained with a CD44 antibody. OPN deposition within the multicellular bulk was accompanied by CD44 enrichment. Bars: (A) 30 μm; (B) 40 μm; (C) 40 μm; 4 μm; (D) 50 μm; (E) 50 μm.

We have highlighted above that invadopodia assembly in GSCs depends on ECM signaling through CD44 and that in total absence of ECM signals (PLL-substrates) GSCs are devoid of invadopodia. Bright-field images showed that GSCs adhered efficiently (due to electrostatic interactions) on PLL (after 2 h incubation) and displayed a rounded morphology (Supplementary Figure 2A). However, a striking change of their morphology was observed when GSCs were left on PLL for 24 h. GSCs passed from a rounded to a more elongated shape (Figure 4A and Supplementary Figure 2A). Immunostaining with cortactin revealed that these elongated GSCs presented invadopodia puncta (Figure 4A). Z-stack imaging further confirmed their columnar nature (Supplementary Figure 2B). Thus, it seems that GSCs can bypass the inhibitory effect of PLL and form invadopodia. Since in GSCs invadopodia assembly depends on CD44 signaling, we explored which extracellular stimuli presented on PLL over time activates the CD44 receptor and restores invadopodia formation. A previous study showed that seeding of WIP^–\–^ (WASP-interacting protein) osteoclasts on OPN rescued podosome formation [25]. In addition, GSCs highly expressing CD44 inhabit in perivascular regions enriched in OPN ligands [9]. To address the hypothesis of an insoluble matrix protein on PLL, GSC-GFP cells incubated for 24 h on PLL were stained for secreted OPN ligands (cells not permeabilized). Interestingly, we observed that OPN positive staining was present in the extracellular space where GFP-GSCs adhered (Figure 4B). Further z-section imaging clearly demonstrated OPN enrichment to the extracellular space underneath the cells (Figure 4C). In addition, we found that GSCs secrete OPN within the multicellular sphere (in non-adherent conditions) since GSC-GFP spheres plated on matrigel-coated coverslips and stained with an OPN antibody showed a profound accumulation of OPN into the multicellular bulk (Figure 4D). Accordingly, GSC-GFP cells cultured as spheres highly expressed CD44 within the bulk following OPN localization pattern (Figure 4E). Strong expression of CD44 within the spheres was accompanied by invadopodia formation as revealed by cortactin-puncta formed on cells tightly compacted into the GFP-spheres (Supplementary Figure 2C). These results suggest that GSCs could provide OPN ligands triggering CD44 activation and invadopodia formation.

### GSCs alone or in cooperation with ECs can contribute to GBM vascularization

**Figure 5:**
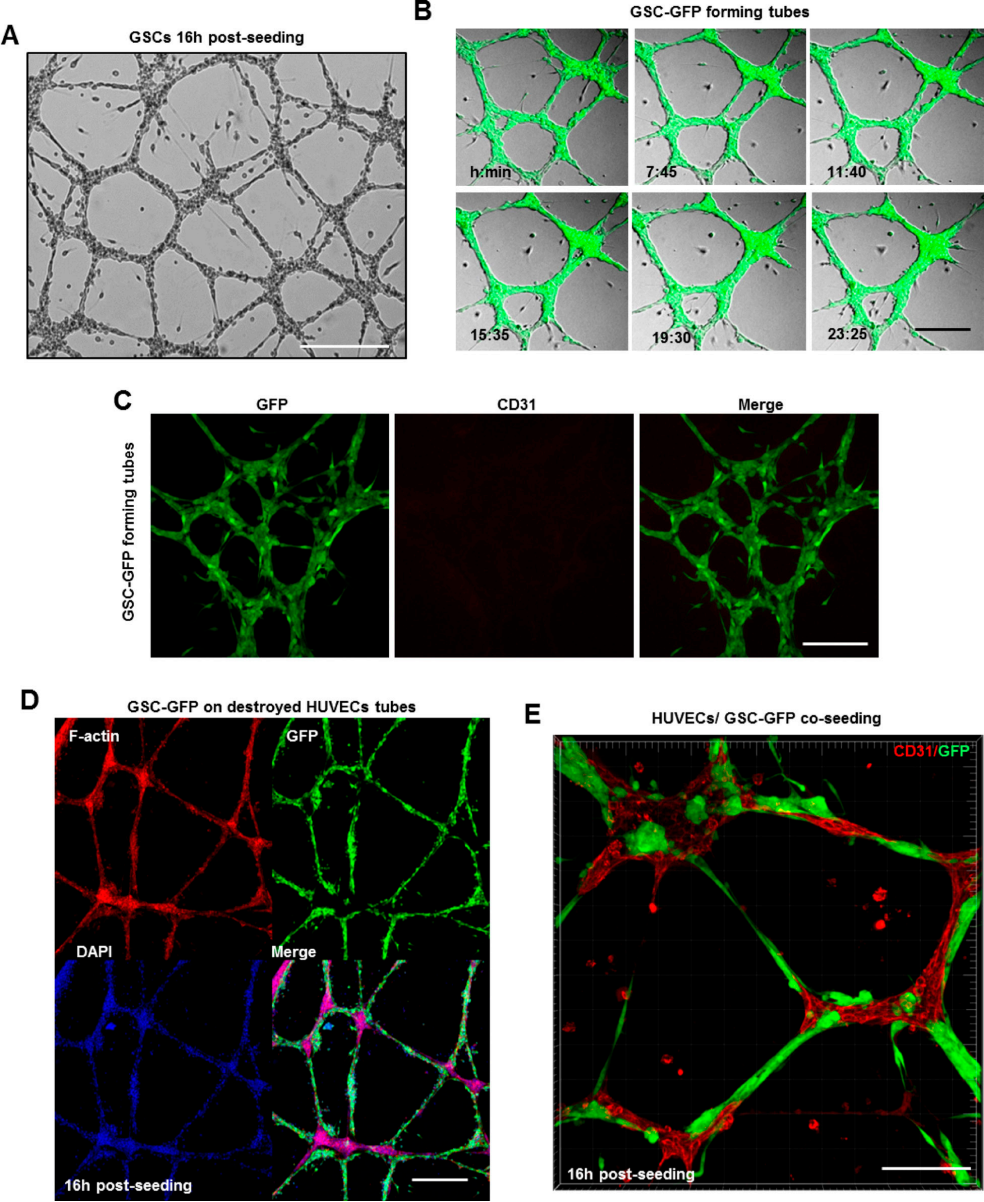
GSCs alone or in cooperation with ECs can contribute to GBM vascularization (**A**) GSCs formed vascular networks reminiscent of normal endothelium over the gelled matrix. (**B**) Representative images extracted from a time series of GSC-GFP cells forming tubes over the gelled matrix. (**C**) GSC-derived tubes were fixed and stained with a CD31 antibody (red). GSC cells did not express the endothelial marker CD31. (**D**) Representative confocal images of GFP expressing GSCs plated on damaged HUVEC-derived tubes and stained for DAPI (blue) and rhodamine phalloidin (red). GSC-GFP cells efficiently restored the destroyed tubes around the HUVEC-remaining branch points. (**E**) A mixed population of GSC-GFP and HUVEC cells was seeded on a gelled substrate in serum-free neurobasal medium. To discriminate GSCs (green) from HUVECs cells, the tubes were fixed and stained with a CD31 antibody (red). GSCs physically interacted with HUVECs and formed intact vascular tubes. Bars: (A) 100 μm; (B) 50 μm; (C) 50 μm; (D) 50 μm; E) 100 μm.

GBMs are lethal brain tumors that exhibit extensive vasculature. Until 2010, it was strongly believed, that tumor vasculature arises by sprouting of pre-existing brain capillaries [19]. However, recent studies revealed that GSCs are capable of differentiation into ECs in order to support tumor vascularization [20–22]. Thus, we examined whether GSCs can contribute to tumor vasculature by forming vascular tubes on a gelled basement matrix (tube formation assay) [26]. First, we assessed the ability of human umbilical vein cells (HUVECs) to form tubes. HUVECs plated on the gelled matrix formed immature capillary-like structures within 3 h which matured into normal tubes 16 h post-seeding (Supplementary Figure 3A). Z-stack analysis of tubes immunostained with the endothelial marker CD31 confirmed HUVECs capacity to form tubular networks (Supplementary Figure 3B). In parallel, dissociated GSC-GFP cells were seeded at the same density over the gelled matrix. Surprisingly, bright field images revealed that even in the absence of serum and VEGF, GSCs formed tubes reminiscent of normal endothelium (Figure 5A). To follow GSCs behavior on the gelled matrix, time-lapse imaging was performed 3 h after GSCs plating for an additional recording time of ∼24 h. During the first 3 h, the cells attached, migrated toward each other and formed tubes. Both bright field and fluorescence imaging showed that GSC-derived tubes were very dynamic structures and that several parameters such as tubes length, number and thickness changed due to cells displacement along the tubes (Figure 5B and Supplementary Video 1). Interestingly, we observed that although GSCs formed tubes, they did not express CD31 marker (Figure 5C).

It is important to note that GSC-tubes resisted over time (∼27 h) while HUVEC-derived tubes began to disrupt after 17–18 h and only the branch points remained intact [26]. To address GSCs ability to restore damaged HUVEC-tubes, GSC-GFP cells were plated on a gelled matrix of remaining HUVEC-aggregates at the branch points. Surprisingly, GSCs migrated, aligned to each other and efficiently restored the damaged tubes (Figure 5D). Given that GSCs were capable to repair destroyed tubular networks, we questioned whether a mixed population of GSC-GFP and HUVEC cells could generate capillary-like networks. Indeed, bright-field images confirmed our hypothesis, revealing the presence of an intact tubular network (Supplementary Figure 3C). To discriminate GSCs from HUVECs cells and in order to examine the participation of each population in tubes formation we conducted immunofluorescence microscopy using the CD31 marker. GSCs physically interacted with HUVECs along the tubes and formed GFP^+^ spherical aggregates that were deeply embedded into the sprouting points mainly composed of HUVECs (Figure 5E). Thus, these results suggest that GSCs alone or in cooperation with normal ECs can contribute to GBM vascularization.

### Functional invadopodia formed in GSCs drive *in vitro* tubes assembly

**Figure 6:**
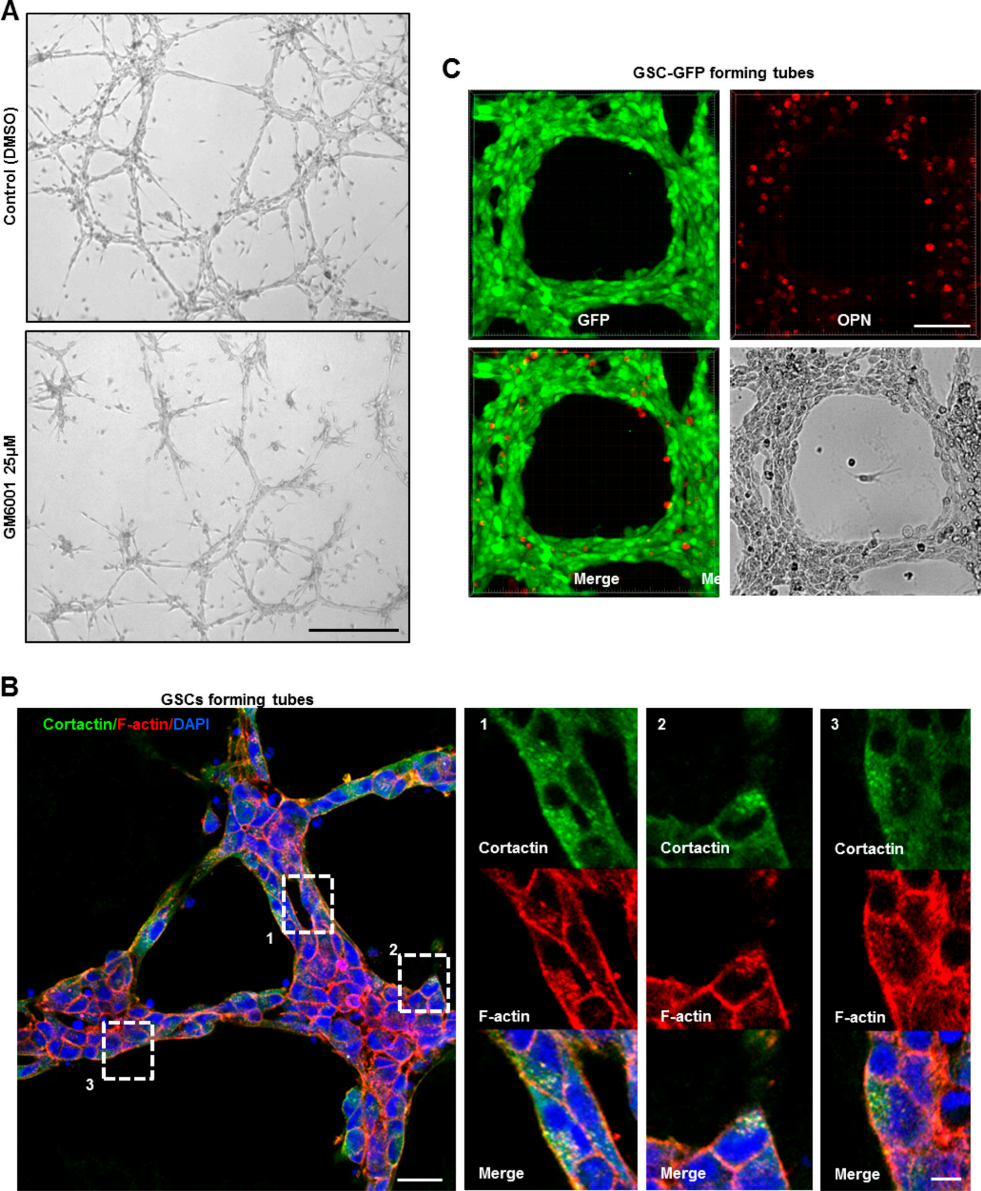
Functional invadopodia formed in GSCs drive *in vitro* tubes assembly (**A**) Tube formation assay of GSCs treated or not with 25 μΜ of the MMP inhibitor GM6001 as the cells were plated on the gelled matrix. GM6001 addition dramatically impacted on GSCs capacity to form intact tubes relative to DMSO-treated cells. (**B**) Intact GSC-GFP-derived tubes were fixed and stained with cortactin (green), rhodamine phalloidin (red) and DAPI (blue). Boxed regions provide a higher magnification of F–actin and cortactin positive dot-like invadopodia structures formed on GSCs tightly compacted into the tubes. (**C**) GSC-GFP cells aligned into tubes were fixed and stained for secreted OPN ligands. OPN staining (red) was present to the extracellular space where GSCs adhered and formed the tubular structures. Bars: (A) 100 μm; (B) 30 μm; 5 μm; (C) 50 μm.

Angiogenesis represents a multi-step process that requires migration/invasion of the ECs across the basement membrane into the surrounding tissue. To enable invasion, ECs pass this physical barrier by secreting proteolytic enzymes such as MMPs [27–29]. Several studies have previously demonstrated the vital role of MMPs during angiogenesis *in vitro* [30–32]. To determine the role of proteases in our *in vitro* angiogenesis system, GSCs were treated with 25 μΜ of the MMP inhibitor (GM6001) as the cells were plated on the gelled matrix. An inhibitory effect on GSCs capacity to form tubes was observed in the presence of GM6001 relative to control cells (DMSO) (Figure 6A). Since MMPs degradative action is mainly restricted to invadopodia sites, we hypothesized that invadopodia-mediated matrix degradation is responsible for tubes assembly [13, 14]. To test this hypothesis, GSC-GFP cells forming tubes were stained for cortactin. Interestingly, careful examination of magnified images of GSC-derived tubes revealed zones where cortactin accumulated at dot-like invadopodium-puncta (Supplementary Figure 4A). To further confirm invadopodia presence, double immunostaining with cortactin and phalloidin was performed (Figure 6B). Indeed, in all micrographs we detected F-actin and cortactin positive dot-like invadopodia. At the molecular level, tube formation over the gelled matrix was accompanied by an increase in the protein levels of invadopodia components such as cortactin and CD44 relative to GSCs lying on matrigel as analyzed by Western blotting. A similar augmentation in paxillin protein levels was also observed (Supplementary Figure 4B).

We have shown above that GSCs secreted on PLL OPN ligands that triggered invadopodia restoration through CD44-mediated signaling. Therefore, it was tempting to explore whether GSCs secrete OPN during tubes assembly. For that purpose, GSCs aligned into tubes were fixed and stained for secreted OPN ligands (cells not permeabilized). As shown in Figure 6C positive OPN staining was apparent to the extracellular space where GSCs adhered and formed the tubular network. OPN deposition on the gelled matrix could also explain the increased levels of CD44 in GSCs forming tubes as compared to cells seeded on matrigel-coated substrates (Supplementary Figure 4B).

**Figure 7:**
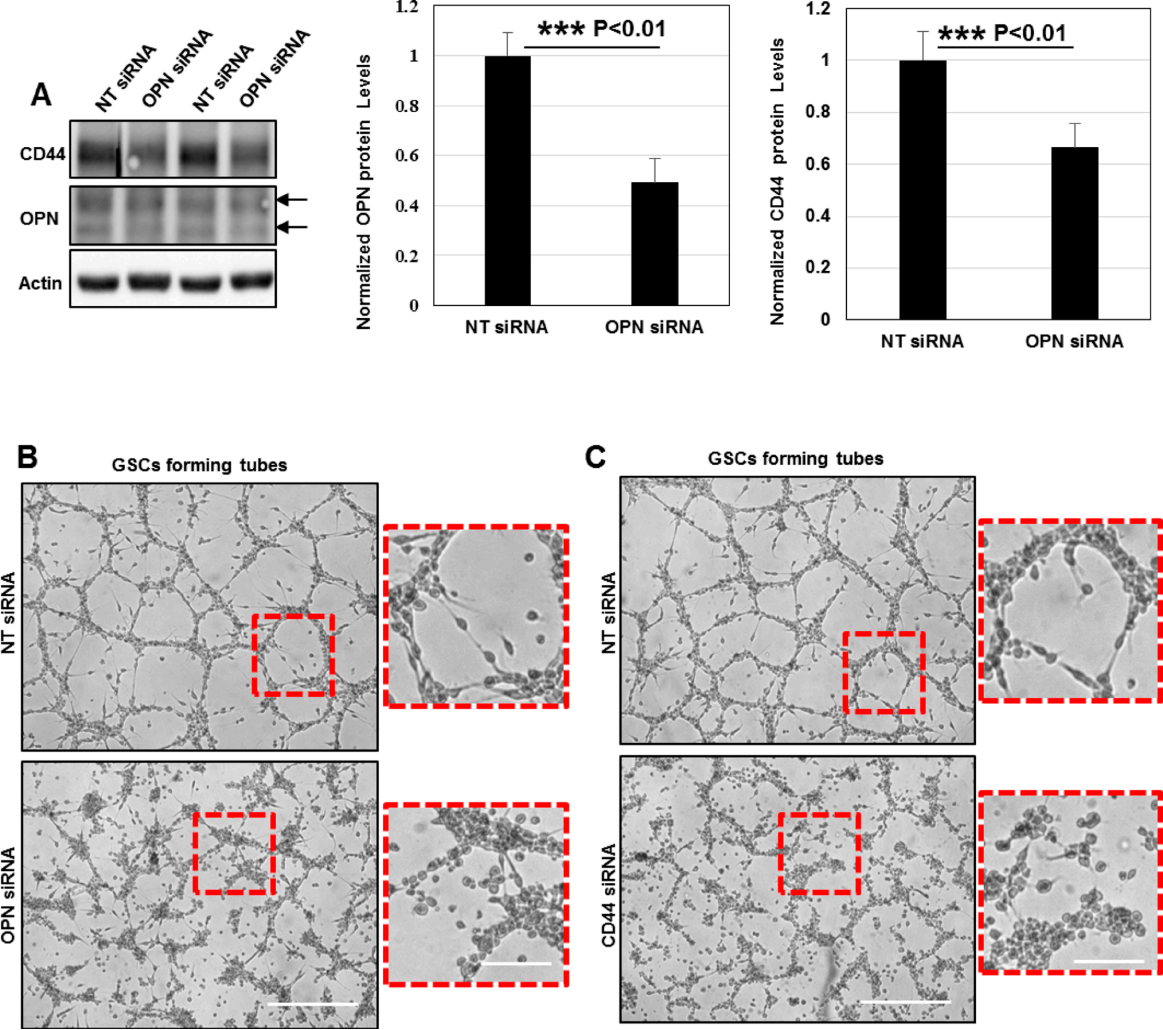
OPN deposition and CD44 signaling promote the formation of functional invadopodia in GSCs during angiogenesis *in vitro* (**A**) Cell lysates of GSCs transfected with NT- or OPN- siRNA were analyzed by Western blotting and probed for CD44, OPN and Actin. SiRNA silencing strategy was efficient leading to 50% decrease in OPN protein levels; ^***^P < 0.01 (*n =* 3). OPN rapid depletion strongly impacted on CD44 protein levels suggesting a functional crosstalk between these proteins ^***^P < 0.01 (*n =* 3). (**B**) NT- or OPN- siRNA KD GSCs were seeded on gelled substrates and allowed to form capillary-like structures for 16 h. OPN inhibition decreased GSCs angiogenic capacity. Boxed regions provide a higher magnification of OPN- and NT- siRNA transfected GSCs. OPN-KD cells exhibited a rounded morphology and were totally devoid of cellular projections compared to control GSCs. (**C**) NT- or CD44- siRNA KD GSCs were seeded on gelled substrates and allowed to form capillary-like structures for 16 h. CD44 depletion dramatically affected cells capacity to form vascular tubes. Boxed regions provide a higher magnification of CD44- and NT- siRNA transfected GSCs. CD44- siRNA KD GSCs acquired a rounded shape and were devoid of cellular projections compared to control GSCs. All graphs presented as means ± SD. Differences with a probability level *P* < 0.05 were considered significant in one-way ANOVA. Bars: (B) 100 μm; 50 μm; (C) 100 μm; 50 μm.

The subsequent question was to determine whether OPN depletion could affect GSCs angiogenic capacity. For that purpose, we used a siRNA-mediated knockdown (KD) to induce OPN silencing in GSC cells. SiRNA-targeting was efficient leading to 50% decrease in OPN protein levels. Surprisingly, we observed that OPN deficiency strongly downregulated CD44 protein levels suggesting that a functional crosstalk between OPN-ligands and CD44 receptor exist in GSCs (Figure 7A). Additionally, OPN knockdown prevented GSCs adhesion/migration on the gelled substrates and inhibited tubes assembly (Figure 7B).

Since OPN signals via CD44 receptor for invadopodia formation we examined the effects of a CD44-mediated KD in *in vitro* angiogenesis assays. In the same manner as OPN depletion CD44 silencing blocked GSCs invasive migration and subsequently tubes formation (Figure 7C). Taken these data under consideration, we conclude that OPN-CD44 signaling promote the formation of fully functional invadopodia in GSCs which drive pathological angiogenesis.

### LIMKs activities are upregulated and highly required for GSCs invasive migration during tubes assembly

**Figure 8:**
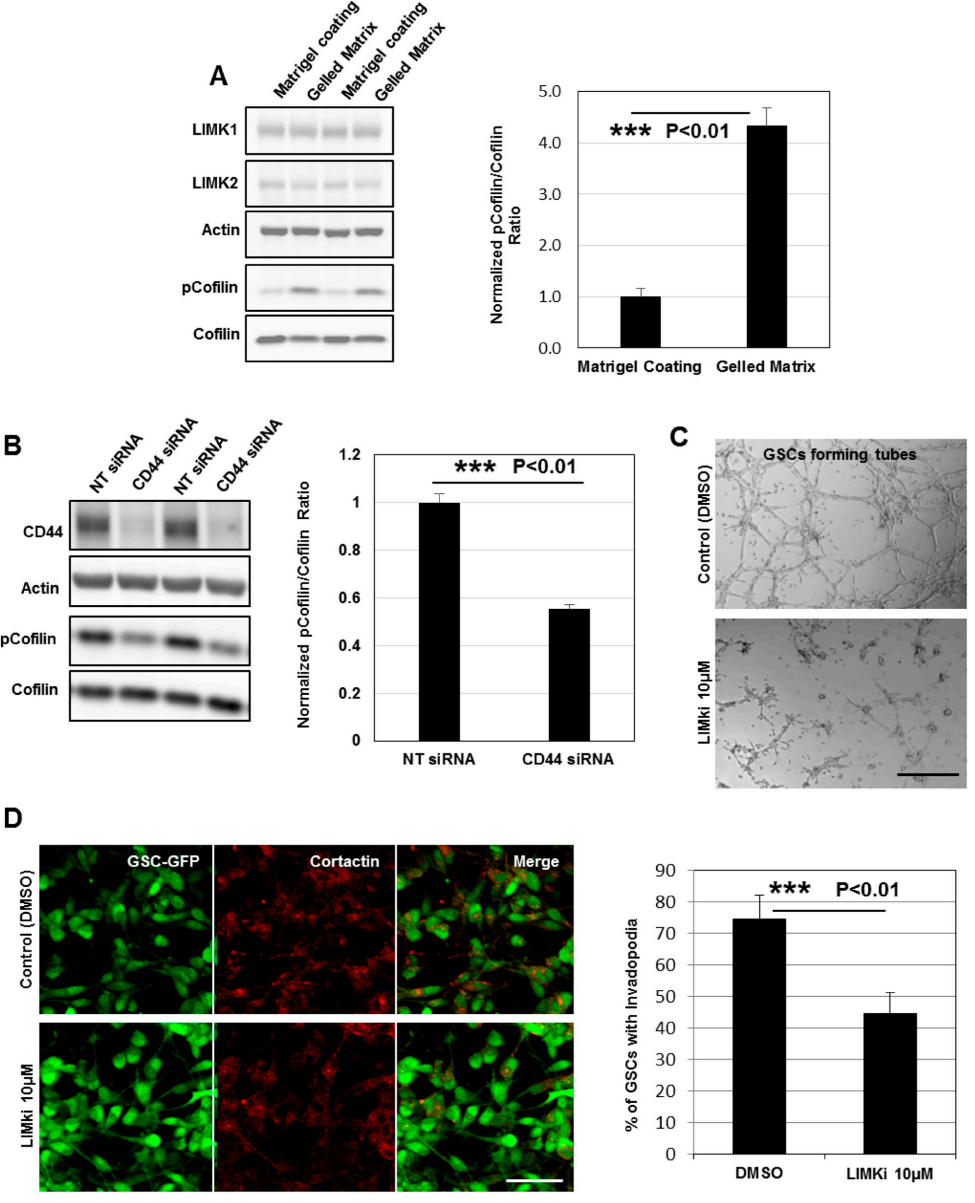
LIMKs activities are upregulated and highly required for GSCs invasive migration during tubes assembly (**A**) Cell lysates of GSCs seeded on matrigel-coated or gelled matrix substrates were analyzed by Western blotting and probed for LIMK1, LIMK2, cofilin, pcofilin and Actin. Quantification of the pcofilin/cofilin ratio showed robust up-regulation of LIMKs activities (3-fold) in GSCs forming tubes; ^***^P < 0.01 (*n =* 4). (**B**) Cell lysates of GSCs transfected with NT- or CD44- siRNA were analyzed by Western blotting and probed for CD44, cofilin, pcofilin and Actin. Quantification of the pcofilin/cofilin ratio showed robust down-regulation of LIMKs activities upon CD44 rapid depletion ^***^P < 0.01 (*n =* 3). (**C**) LIMKi addition dramatically impacted on GSCs capacity to form intact tubes. (**D**) LIMKs inhibition significantly decreased GSCs ability to form invadopodia; ^***^P < 0.01 (*n =* 3). All graphs presented as means ± SD. Differences with a probability level P < 0.05 were considered significant in one-way ANOVA. Bars: (C) 100 μm; (D) 50 μm.

As the expression of LIMK1&2 and their substrate cofilin is upregulated in GBM [33], we measured their protein expression by immunoblotting in GSCs relative to HUVECs. The data showed that the endogenous level of LIMK1 (but not LIMK2 and cofilin) was at least 100% higher in GSCs compared to HUVECs (Supplementary Figure 5A). Interestingly, we found also a robust up-regulation of LIMKs activities (based on cofilin phosphorylation status) in GSC-forming tubes over gelled substrates compared to GSCs seeded on matrigel-coated surfaces. Indeed, by quantifying the pcofilin/cofilin ratio in both conditions we observed a 3-fold increase in GSC-forming tubes indicating that LIM kinases may have an important role during angiogenesis (Figure 8A). Since LIMKs activities and CD44 signaling are both upregulated during tubes assembly we examined whether CD44-transmitted signals might activate LIM kinases. To address this question LIMKs activities (based on cofilin phosphorylation status) were determined in NT- and CD44- siRNA treated GSCs. Interestingly, we found a robust down-regulation of LIMKs activities upon CD44 inhibition (∼45% decrease in pCofilin/Cofilin ratio) suggesting that OPN-CD44 signaling exerts its effects by regulating downstream LIMKs (Figure 8B).

To further examine the contribution of LIMKs to GSCs angiogenic capacity, we tested a selective inhibitor of both LIMK1&2 also referred as LIMKi. GSCs treatment with LIMKi significantly inhibited cofilin phosphorylation at 10 μΜ (Supplementary Figure 5B). LIMKs inhibition also impaired GSCs capacity to form tubes compared to control cells (DMSO) (Figure 8C). To determine if GSCs incapacity to form vascular channels is due to invadopodia disassembly, GSC-GFP cells treated with LIMKi or DMSO were stained for cortactin. Notably, we observed that LIMKi treatment significantly affected GSCs capacity to form invadopodia, which suggests that LIM kinases activity is necessary for invadopodia formation (Figure 8D). Since angiogenesis is based on cells migratory capacities, we next assessed the effects of LIMKi on the migration of GSCs on two-dimensional (2D) matrigel-coated surfaces. GSC-spheres pretreated with LIMKi (10 μΜ) or DMSO (for 10 h in non-adherent cultures) were subcultured over the 2D substrates and GSCs migration tendency was evaluated by quantifying the number of filopodium-like protrusions (FLP) formed per sphere. At 4 h post-seeding, control spheroids extended numerous FLP whereas LIMKi addition had a thorough effect on FLP abundance (Supplementary Figure 5C). We then analyzed LIMKi effects on GSCs invasive migration using a HUVEC-based transmigration system. Pretreated GSC-spheres were seeded on top of the HUVECs confluent monolayer in inhibitor-free medium. Control-spheroids adhered on top of HUVECs and GSCs progressive unloading over the monolayer disrupted HUVECs cell-cell contacts creating thus an invasion area (Supplementary Figure 5D and Supplementary Video 2). In contrast, LIMKi exerted a strong inhibitory effect on GSCs transmigration capacities (Supplementary Figure 5E). Together, these findings suggest that LIM kinases are important for both the formation of invadopodia and GSCs invasive migration during tubes assembly.

Previous studies highlighted that pharmacological inhibition of LIMKs could have antitumor effects [34–36]. Therefore, it was challenging to test the impact of the LIMKi in GSCs growth. We compared spheres size generated by GSCs treated with LIMKi or DMSO. At day 15 post-seeding, LIMKi-treated cells generated spheroids which were smaller in size as evaluated by morphometric parameters such as the surface area and spheres perimeter (Supplementary Figure 6A). Indeed, cell proliferation as assessed by Ki67 staining and quantification was significantly decreased in LIMKi-treated GSCs (Supplementary Figure 6B). Collectively, these data suggest that LIMKs activities are upregulated during tubes assembly and are highly required for GSCs invasive migration and growth.

## DISCUSSION

Several studies over the past 10 years have demonstrated that GSCs inhabit within protective vascular niches where physical interactions and niche-derived factors maintained GSCs stemness [8, 9]. Moreover, other studies have reported the presence of cancer cells in the walls of tumor blood vessels which were devoid of endothelial cells [37, 38]. Recently, new data revealed that GSCs can actively participate in tumor vascularization by generating endothelial cells [20–22]. Herein we provide evidence that GSCs derived from an adult GBM-patient represent a source of angiogenic players exerting important roles during angiogenesis *in vitro*. GSCs angiogenic capacity is mediated by invadopodia and depends on their degradative activity. Finally, tubes assembly relies on LIMKs activities that are highly required for GSCs invasive migration over the gelled substrates.

Invadopodia of tumor cells and podosome-like structures in normal and Src-transformed cells collectively known as invadosomes have been widely recognized as invasive structures. Both podosomes and invadopodia appear as actin-rich puncta on the lateral cellular that localize intense ECM degrading activity. Invadopodia formation is generally associated with cancer cells and is often related to their invasive and metastatic potentials [13, 14, 16, 17]. Although invadopodia formation occurs in highly metastatic cancer cells, little evidence exists for their assembly in GBM cells. Herein we confirmed invadopodia presence in GSCs as membrane protrusions extending deeply into FITC-gelatin layers where ECM degradation also occurred (Figure 1). While podosomes and invadopodia display some common characteristics in architecture, composition and function, several differences have been noted [16, 39, 40]. For example, podosomes assembly occurs only in adherent cells explaining thus the importance of a cross-talk between the matrix and surface receptors. Indeed, podosomes are enriched in adhesive receptors such as integrins or CD44 which mediate the close contact with the surrounding matrix [25, 40–43]. In contrast, although invadopodia seem to closely interact and receive signals from ECM components, the question of whether these structures bind to the ECM remains obscure [44]. Comparisons between GSCs seeded on matrigel- or PLL-coated substrates permitted us to show that invadopodia assembly in GSCs is triggered by ECM signals transmitted via CD44 receptors (Figure 2A and 2B). The potential role of transmembrane receptors in invadopodia formation was further confirmed by the localization of CD44 within the cores and by the decreased GSCs capacity to form invadopodia when CD44-trasmitted signals were blocked by a siRNA-mediated knockdown (Figure 2D and Figure 3).

GBMs are fatal tumors and striking angiogenesis is one of the pathological hallmarks of this disease. Therefore, antiangiogenic therapies targeting VEGF/VEGFR pathway have been approved for treatment of recurrent GBM. Even though these therapies were well tolerated, tumor progression inevitably occurs. [23, 24, 45]. However, Soda *et al.* highlighted that GBM resistance to antiangiogenic therapies is strongly associated with the presence of GSCs which are able to differentiate into endothelial cells and to drive tumor angiogenesis [20]. Using *in vitro* assays, we showed that GBM-isolated GSCs form tubular networks morphologically comparable with those shown by normal endothelial cells (HUVECs). Interestingly, we observed that GSCs in tubes do not express the endothelial marker CD31 suggesting their incapacity to differentiate into endothelial cells (Figure 5A–5C). However, an alternative mechanism of GBM neovascularization has been described and termed as vascular mimicry. This term was used to describe blood-perfused vascular channels exclusively composed by tumor cells that can mimic endothelial cells function [46–49]. GSCs capacity for vascular mimicry was further confirmed in this study by testing GSCs reaction to destroyed endothelial tubes. GSCs migrated, aligned into tubes and efficiently restored the damaged capillary-like structures around the HUVEC-remaining branch points (Figure 5D). Furthermore, we questioned whether “mosaic tumor vessels” can also be generated *in vitro*. The “mosaic” pattern characterizes vessels in which lumens are composed by both normal ECs and tumor cells lacking endothelial markers [48, 50]. Indeed, in this study we showed that a mixed population of GSCs and HUVECs can generate intact “mosaic” tubular networks composed of thin tubes emanating by bulky branch points. By bright-field images GSCs were indistinguishable from HUVECs and additional immunofluorescence analysis (CD31 and GFP-tumor specific marker) was needed to monitor the participation of each population during tubes formation (Figure 5E and Supplementary Figure 3C).

One of the most interesting points addressed in this study focused on the mechanisms by which angiogenic GSCs adhere, migrate and form capillary-like structures on the gelled matrigel-based matrices. This is the first finding to our knowledge demonstrating that invadopodia assembly and activity in GSCs is highly necessary for *in vitro* angiogenesis. Indeed, we showed that fully functional invadopodia formed in GSCs efficiently remodeled the gelled substrates and permitted tubes assembly (Figure 6A and 6B). Moreover, we highlighted that GSCs aligned into tubes secreted over the gelled substrates OPN ligands which reinforced cells adhesion and invadopodia formation via CD44-mediated signaling (Figure 6C and Figure 7).

Given that aberrant expression of LIM kinases has been implicated in numerous malignancies such as breast tumors and GBMs [33, 36, 51] we investigated the role of these kinases in GBM invasive migration during angiogenesis *in vitro*. In the current study, we provided evidence that during tubes formation, LIMKs activities are upregulated and promote GSCs invasion over the gelled substrates (Figure 8A). Moreover, we showed that OPN-CD44 pathway exerts its effects by influencing LIMKs activities (Figure 8B). Having established that LIMKs signaling plays a key role for tubes assembly we evaluated the efficiency of the LIMK inhibitor LIMKi on GBM invasiveness. We found that LIMKi significantly inhibited cofilin phosphorylation at a therapeutic dose and reduced GSCs migratory and invasive capacities (Supplementary Figure 5). Additionally, we demonstrated that LIMKs inhibition impaired invadopodia formation and subsequently GSCs alignment into vascular tubes (Figure 8C and 8D). Significant decrease in GSCs proliferation (Supplementary Figure 6) was also observed suggesting that LIMK inhibitors such as LIMKi could represent effective agents to target GBM invasiveness and growth.

Taken together, these data provide new insights of GBM microvasculature and suggest that GSCs targeting in combination with anti-VEGF therapy may block tumor progression.

## MATERIALS AND METHODS

### Antibodies and reagents

Mouse monoclonal anti-Cortactin (p80/85, clone 4F11), anti-Nestin and rabbit polyclonal antibody against SOX2 were obtained from Merck Millipore. Mouse monoclonal anti-Paxillin (clone 349) antibody was purchased from BD Biosciences. Rabbit monoclonal anti-LIMK2 (8C11), anti-Cofilin (D3F9), anti-phospho-Cofilin (77G2) and rabbit polyclonal anti-LIMK1 antibodies were purchased from Cell Signaling Technology. A rabbit polyclonal antibody against CD44 (HCAM, [H-300]) and a mouse monoclonal antibody against Osteopontin (OPN, [LFMb-14]) were purchased from Santa Cruz Biotechnology. A rabbit polyclonal antibody against Ki67 (ab15580) was obtained from Abcam. Mouse monoclonal anti-CD31 (PECAM-1, [9G11]) antibody was from R&D Systems. Alexa 546-labeled Phalloidin, Alexa 488-, 555-conjugated secondary antibodies (goat anti-mouse and goat anti-rabbit) were purchased from Life Technologies. Anti-actin mouse monoclonal and (HRP)-conjugated (goat anti-mouse and goat anti-rabbit) antibodies were purchased from Abcam and Cell Signaling Technology respectively.

GM6001 MMP Inhibitor (broad-spectrum inhibitor of MMPs) and LIM Kinase Inhibitor I (LIMKi 3), which inhibits LIMK1&2 kinases were used at 25 μM and 10 μM respectively and were purchased from Calbiochem. Growth Factor Reduced BD Matrigel Matrix and Poly-L-lysine solution were obtained from VWR and Sigma-Aldrich respectively.

siRNAs (ON-TARGETplus) targeting CD44 (L-009999-06), OPN (also referred as SPP1) (L-012558-09) and NT (Non-targeting Pool) (D-001810-10-05) were purchased from Thermo Fisher Scientific and transfected with Lipofectamine RNAiMAX (Invitrogen) following the standard protocol. Knockdown was performed via two rounds of siRNA transfection at 24-h intervals. The following siRNA (Dharmacon) sequences were used: on target Non-targeting Pool (5′-UGGUUUACAUGUCGACUAA-3′, 5′-UGGUUUACAUGUUGUGUGA-3′, 5′-UGGUUUACAUGUUUUCUGA-3′, 5′-UGGUUUACAUGUUUUCCUA-3′); on target-SMART pool CD44 (5′-GAAUAUAACCUGCCGCUUU-3′, 5′-CAAGUGGACUCAACGGAGA-3′, 5′-CGAAGAAGGUGUGGGCAGA-3′, 5′-GAUCAACAGUGGCAAUGGA-3′); on target-SMART pool SPP1 (5′-CCAAGUAAGUCCAACGAAA-3′, 5′-CAUCUUCUGAGGUCAAUUA-3′, 5′-UGAACGCGCCUUCUGAUUG-3′, 5′-GAUGAACUGGUCACUGAUU-3′).

### Cell culture and infection experiments

GSC cell line (GSC-2) used in this study derived from an adult GBM-patient operated in Poitiers University Hospital was characterized and cultured as previously described [52–55]. Briefly, the GBM tumor was washed and mechanically dissociated into single cells. Dissociated cells were cultured in Neurobasal medium (NBE) supplemented with 20 ng/mL of basic fibroblast growth factor (bFGF, Invitrogen), 20 ng/mL of epidermal growth factor (EGF, Invitrogen) and the culture supplements N2 (100×, Invitrogen) and B27 (50×, Invitrogen). GSC cells positive for the surface marker CD133 were isolated by magnetic cell sorting. GSCs were cultured as non-adherent spheroids and at the point spheres augmented in size were enzymatically dissociated using accutase (Merck Millipore). GFP expression to GSCs was performed via infection using the lentiviral vector that encodes the green fluorescent protein TRIP/ΔU3-EF1α-GFP as previously described [52].

To study the potential role of CD44 signaling in invadopodia formation, GSCs were transfected with NT- and CD44- siRNA using Lipofectamine RNAiMAX. Knockdown was performed via two rounds of siRNA transfection at 24-h intervals. NT- and CD44- siRNA transfected GSC-GFP cells were then seeded on matrigel-coated coverslips for 4 h, fixed and stained with phalloidin. Fixed cells were examined using a confocal laser-scanning microscope (IX81; Olympus) equipped with 40× (NA 1.35) UAPO ID/340UV (oil immersion) and 60× (NA 1.4) PLAPO (oil immersion) objectives. The fluorescence images were sampled with FV1000 Viewer software (Olympus). The images were then processed with ImageJ (http://rsb.info.nih.gov/ij/).

For the sphere formation assays, GSC dissociated cells were subcultured (160 cells per cm^2^) in 96-well plates in serum-free neurobasal medium in the presence of LIMKi and vehicle (DMSO) for 15 days. Bright field images of the spheres were taken using an inverted microscope (CKX41; Olympus) equipped with 4× (NA 0.10) PlanC-N, and 10× (NA 0.25) PlanC-N objectives. Images were then sampled with ToupView software (ToupTek Photonics). Morphometric parameters such as spheres surface area and perimeter were quantified using ImageJ.

HUVEC cells were purchased from Lonza and cultured in EBM-2 medium (Lonza) supplemented with HUVEC growth factors (Lonza). Only early passages of HUVECs (between 4 and 6) were used. For the tube formation assay, HUVECs were seeded at a density of 8.5 × 10^4^ cells/cm^2^ in FluroDish™ culture dishes (35 mm, World Precision Instruments Inc) in supplemented EBM-2 media over a gelled basement matrix (Matrigel; 3 mg/mL) and allowed to form capillary-like structures for 16h. In the same manner, NT-, CD44- and OPN- siRNA transfected GSCs were seeded at a density of 8.5 × 10^4^ cells/cm^2^ in FluroDish™ culture dishes (35 mm, World Precision Instruments Inc) in supplemented NBE media over a gelled basement matrix (Matrigel; 3 mg/mL) and allowed to form capillary-like structures for 16 h. Bright field images of the vascular tubes were taken using an inverted microscope (CKX41; Olympus) equipped with 4× (NA 0.10) PlanC-N, and 10× (NA 0.25) PlanC-N objectives. Images were then sampled with ToupView software (ToupTek Photonics) and processed with ImageJ (http://rsb.info.nih.gov/ij/).

### Immunofluorescence microscopy and live-imaging

For immunofluorescence analysis, GSCs, GSC-spheres and HUVECs seeded on glass coverslips (coated with Matrigel or Poly-L-lysine) or gelled substrates were fixed with 4% paraformaldehyde in PBS, permeabilized or not (0.2% Triton X-100) and blocked in 4% bovine serum albumin (BSA) in PBS. Cells were then incubated for 1 h at room temperature (RT) with primary antibodies and rinsed in PBS. Secondary antibodies were then added for 1 h at RT. Coverslips were permanently mounted in PVA-DABCO. Fixed cells were examined using a confocal laser-scanning microscope (IX81; Olympus) equipped with 10× (NA 0.4) UPLAPO, 20x (NA 0.70) UPLAPO, 40× (NA 1.35) UAPO ID/340UV (oil immersion) and 60× (NA 1.4) PLAPO (oil immersion) objectives. The fluorescence images were sampled with FV1000 Viewer software (Olympus). The images were then processed with ImageJ (http://rsb.info.nih.gov/ij/) and Imaris v7 (Bitplane) software.

For live imaging of the tube formation assays, GSC-GFP cells were seeded over a gelled basement matrix in FluroDish™ culture dishes and allowed to form capillary-like structures for 3 h before imaging. Dishes were placed on a heated 37° C stage and imaged with a spinning disk confocal microscope (IX81-ZDC; Olympus) equipped with a 20× (NA 0.75) UPLSAPO objective (Supplementary Video 1). Fluorescence images were sampled with FluoView software (Olympus) using an interval time of 5 min for a total recording of 23,5 h. Time-lapse movies were then processed with ImageJ software.

To monitor GSCs transmigration capacities on HUVEC-based monolayers, GFP-GSC spheroids were seeded on top of confluent monolayer and live imaging was performed with a spinning disk confocal microscope (IX81-ZDC; Olympus) equipped with a 20× (NA 0.75) UPLSAPO objective (Supplementary Video 2). Fluorescence images were sampled with FluoView software (Olympus) using an interval time of 5 min for a total recording of 21,5h. Time-lapse movies were then processed with ImageJ software.

### Extracellular matrix degradation assays

The QCM™ Gelatin Invadopodia Assay (Green) (Merck Millipore) was used to assess invadopodia matrix degradation activity following manufacturer’s instructions. Briefly, glass coverslips were sterilized in 70% ethanol for 15 minutes at RT. Air-dried coverslips were then incubated with Poly-L-lysine solution for 20 min at RT. Washed coverslips were then incubated for 15 min with a Glutaraldehyde solution. After Glutaraldehyde incubation glass coverslips were inverted into droplets containing a mixture of Fluorescein-conjugated/unlabeled gelatin to a ratio of 1:9 for 10 min. Coverslips were subsequently incubated in 5 mg/ml NaBH4 for 15 min, rinsed in PBS and sterilized in 70% ethanol for 15 minutes at RT. GSCs were dissociated and seeded over the coverslips for 18 h (2.5 × 10^4^/per coverslip). The cells were fixed and observed with a confocal laser-scanning microscope (IX81; Olympus) equipped with a 40× (NA 1.35) UAPO ID/340UV (oil immersion) or a 60× (NA 1.4) PLAPO (oil immersion) objective. The fluorescence images were sampled with FV1000 Viewer software (Olympus). Image analysis was performed using ImageJ and Imaris v7 software.

### Western blotting

Whole cell lysates were prepared in a cold RIPA buffer. 30 μg of proteins from each cell lysate was subjected to SDS-polyacrylamide gel electrophoresis (PAGE), transferred to nitrocellulose membranes (Bio-rad), and probed with primary antibodies. HRP-conjugated goat anti-mouse or goat anti-rabbit secondary antibodies were detected by enhanced chemiluminescence (Clarity™ Western ECL Substrate, Bio-rad) with Luminescent Image Analyzer LAS-3000 (FUJIFILM). Proteins were quantified by the ImageJ software and actin was used as a loading control. Each quantified protein was normalized to actin.

### Statistical analysis

Quantitative data (morphometric parameters such as spheres surface area/perimeter, percentage of Ki67^+^ cells, percentage of GSCs positive for invadopodia and Western blot quantifications) are presented as means ± SD and statistically analyzed with NCSS 2004 software by one-way analysis of variance (ANOVA). Differences with a probability level *P* < 0.05 were considered significant. All graphs include standard deviation error bars.

## SUPPLEMENTARY MATERIALS FIGURES AND VIDEOS







## Abbreviations

GBM: Glioblastoma
GSCs: Glioblastoma Stem Cells
ECs: Endothelial Cells
EGF: Epidermal Growth Factor
bFGF: Basic Fibroblast Growth Factor
VEGF: Vascular Endothelial Growth Factor
VEGFR: Vascular Endothelial Growth Factor Receptor
FLP: Filopodium-like protrusions
HUVECs: Human Umbilical Vein Cells
LIMK1: LIM domain kinase 1
LIMK2: LIM domain kinase 2
MMP: Matrix Metalloproteinase
OPN: Osteopontin
PLL: Poly-L-Lysine
