# Supplementary material for: Functional invadopodia formed in glioblastoma stem cells are important regulators of tumor angiogenesis
Source: Oncotarget. 2018 Apr 17;9(29):20640–57. doi: 10.18632/oncotarget.25045 (PMC5945526; doi:10.18632/oncotarget.25045)
Supplement: Supplementary file 1 [file oncotarget-09-20640-s001.pdf]

Functional invadopodia formed in glioblastoma stem cells are important regulators of tumor angiogenesis

SUPPLEMENTARY MATERIALS

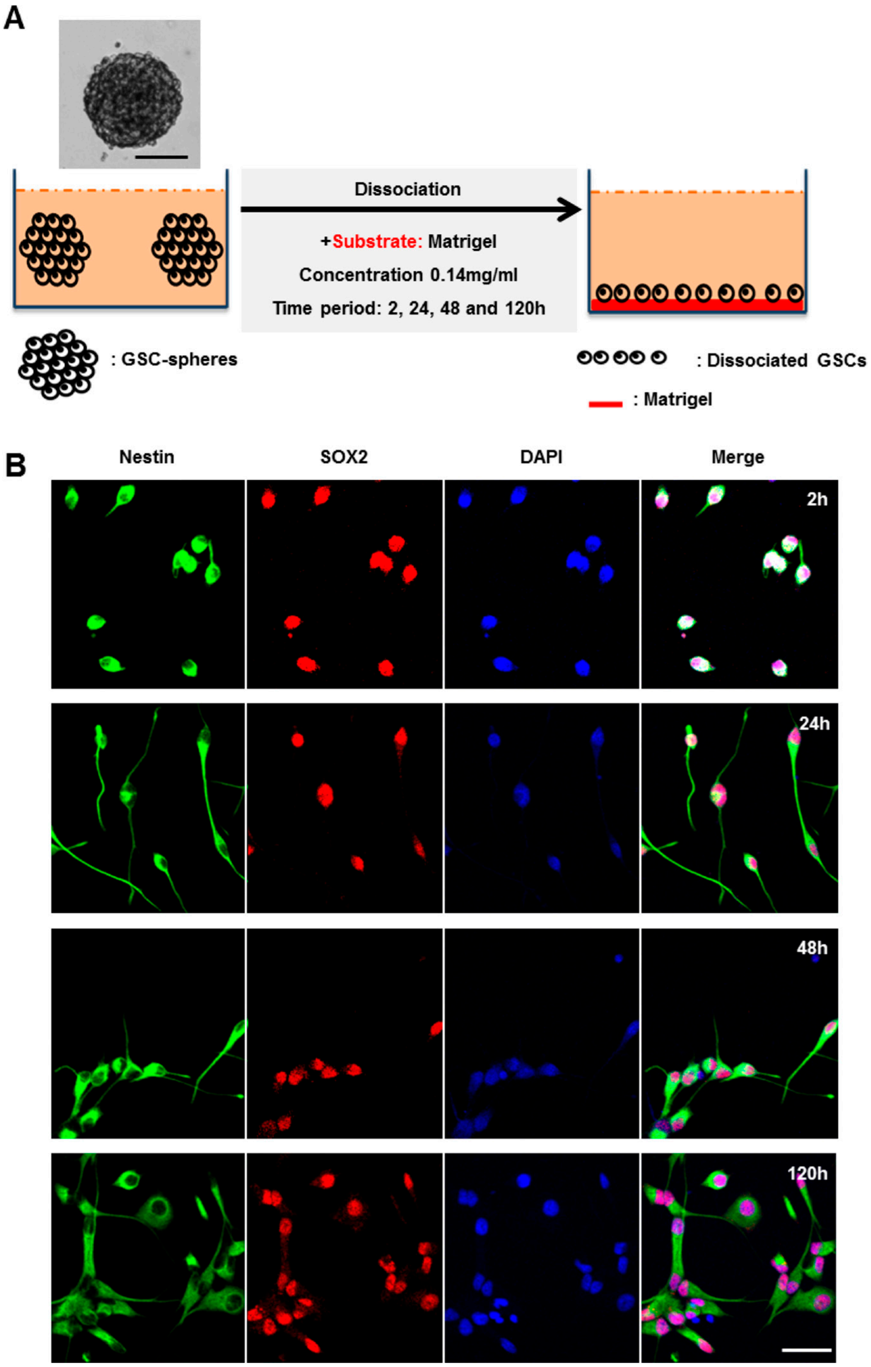

Supplementary Figure 1: GSCs upon adhesion retained their stem-cell phenotype. (**A**) GSCs are maintained in culture in defined serum-free medium and proliferate as non-adherent multicellular spheres. Spheres were dissociated and isolated cells were plated on matrigel-coated substrates. (**B**) GSCs seeded on matrigel substrates for different time periods were fixed and stained for stem-cell markers such as Nestin (green) and SOX2 (red). GSCs adhesion on matrigel does not affect their stem cell phenotype. (A) 30 µm; (B) 20 µm.

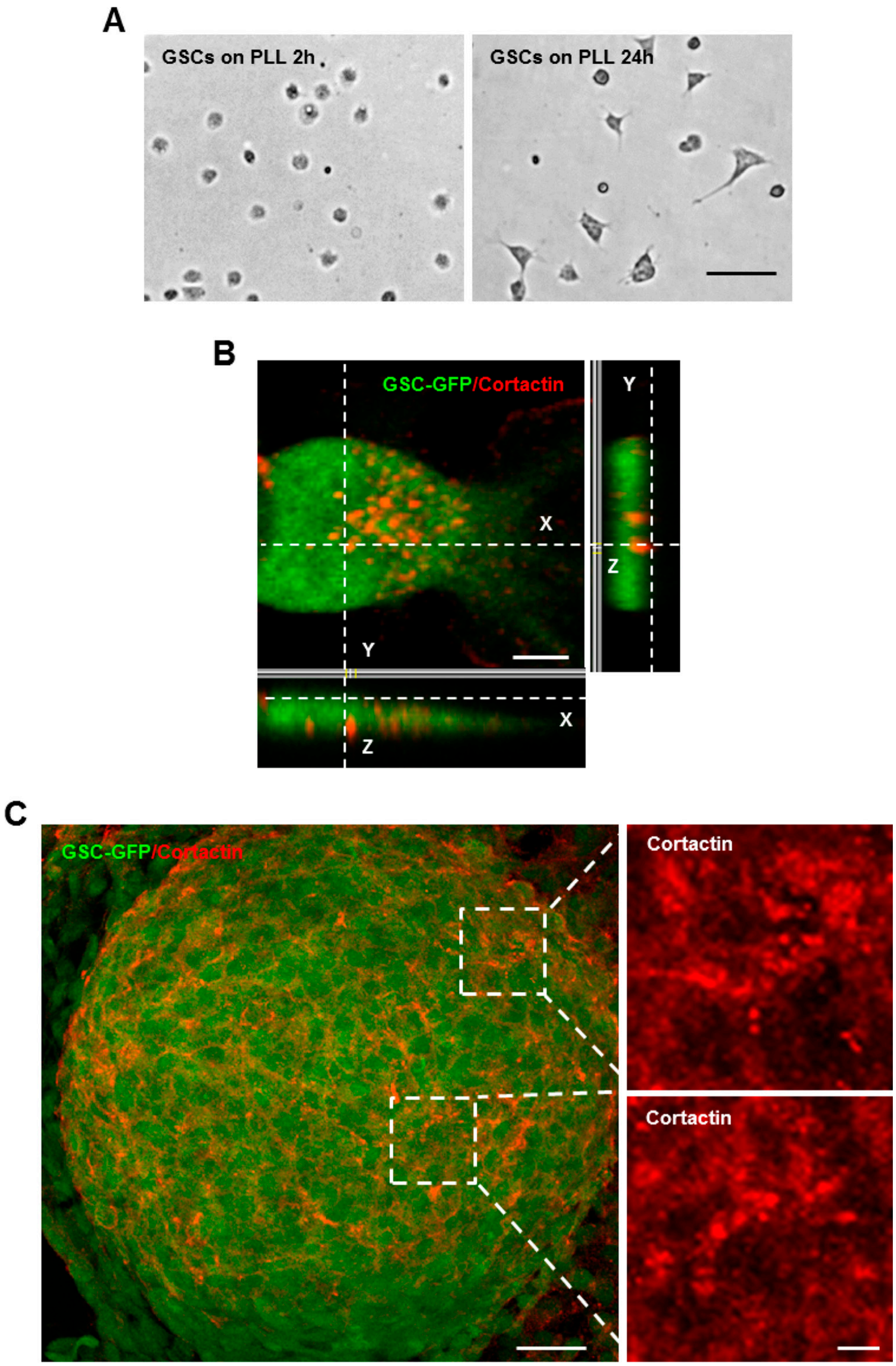

Supplementary Figure 2: OPN deposition controls invadopodia formation. (**A**) Bright filed images of GSCs plated on PLL for different time periods (2 and 24 h). GSCs incubation on PLL for 24 h strongly affected cells morphology. (**B**) GFP-expressing GSCs were cultured on PLL for 24 h and stained with anti-cortactin antibody (red). Z-stack analysis confirmed the presence of numerous cortactin-containing protrusions on the ventral cellular side. (**C**) GFP-expressing spheres were plated on matrigel and stained with cortactin (red). Boxed regions provide a higher magnification of cortactin-containing invadopodia formed on cells tightly compacted into the GFP-spheres. Bars: (A) 50 µm; (B) 30 µm; (C) 50 µm; 5 µm.

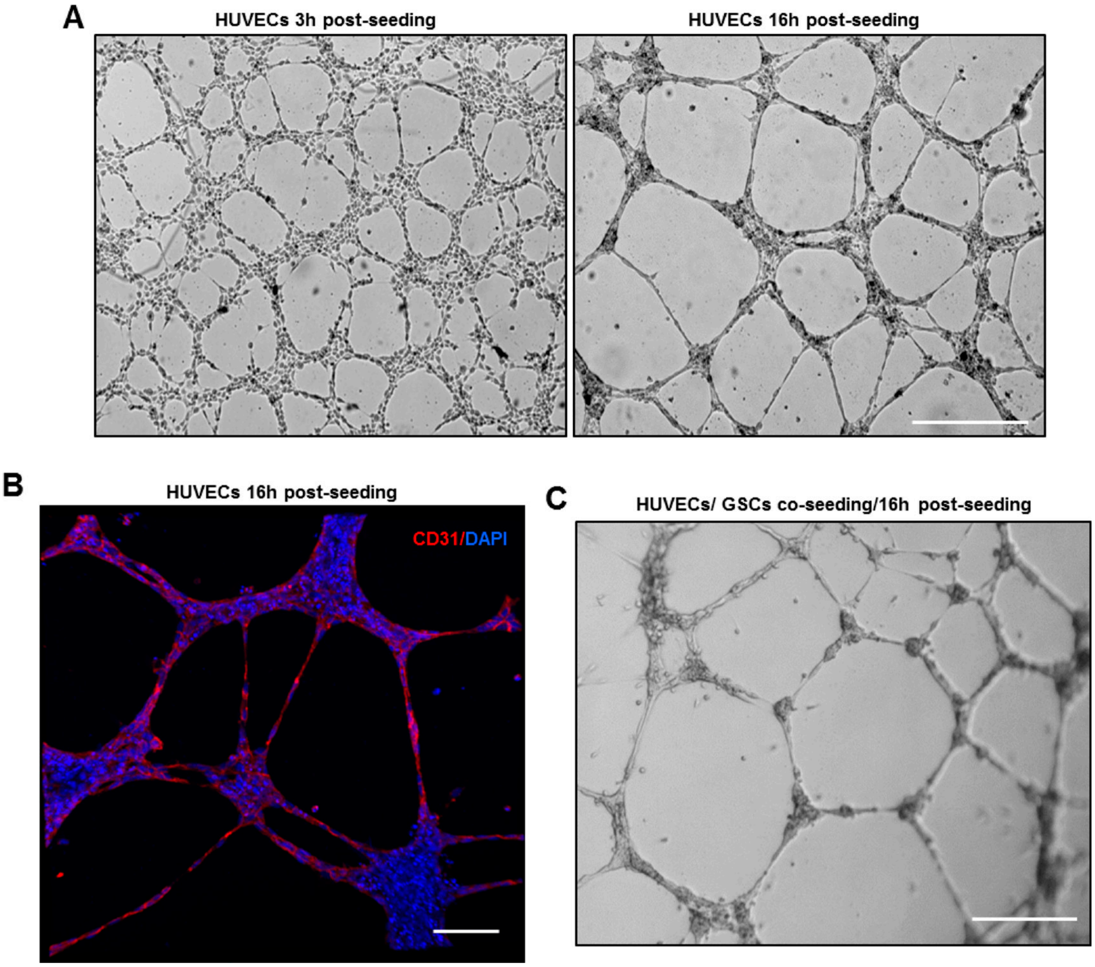

Supplementary Figure 3: Testing HUVECs and GSCs angiogenic capacity. (**A**) Tube formation assay of HUVEC cells cultured over the gelled matrix in EBM-2 medium supplemented with HUVEC growth factors (bFGF, EGF, IGF and VEGF) and 2% serum. HUVECs plated at high density on the gelled matrix aligned into capillary-like structures within 3 h which further matured into normal tubes 16 h post-seeding. (**B**) Representative confocal images of HUVEC-derived tubes formed on the gelled matrix and stained for DAPI (blue) and CD31 endothelial marker (red). (**C**) Bright field images of the mixed population (GSC-GFP/HUVEC cells; ratio 1:1) seeded on the gelled substrate in serum-free neurobasal medium. Bars: (A) 100 μm; (B) 50 μm; (C) 100 μm.

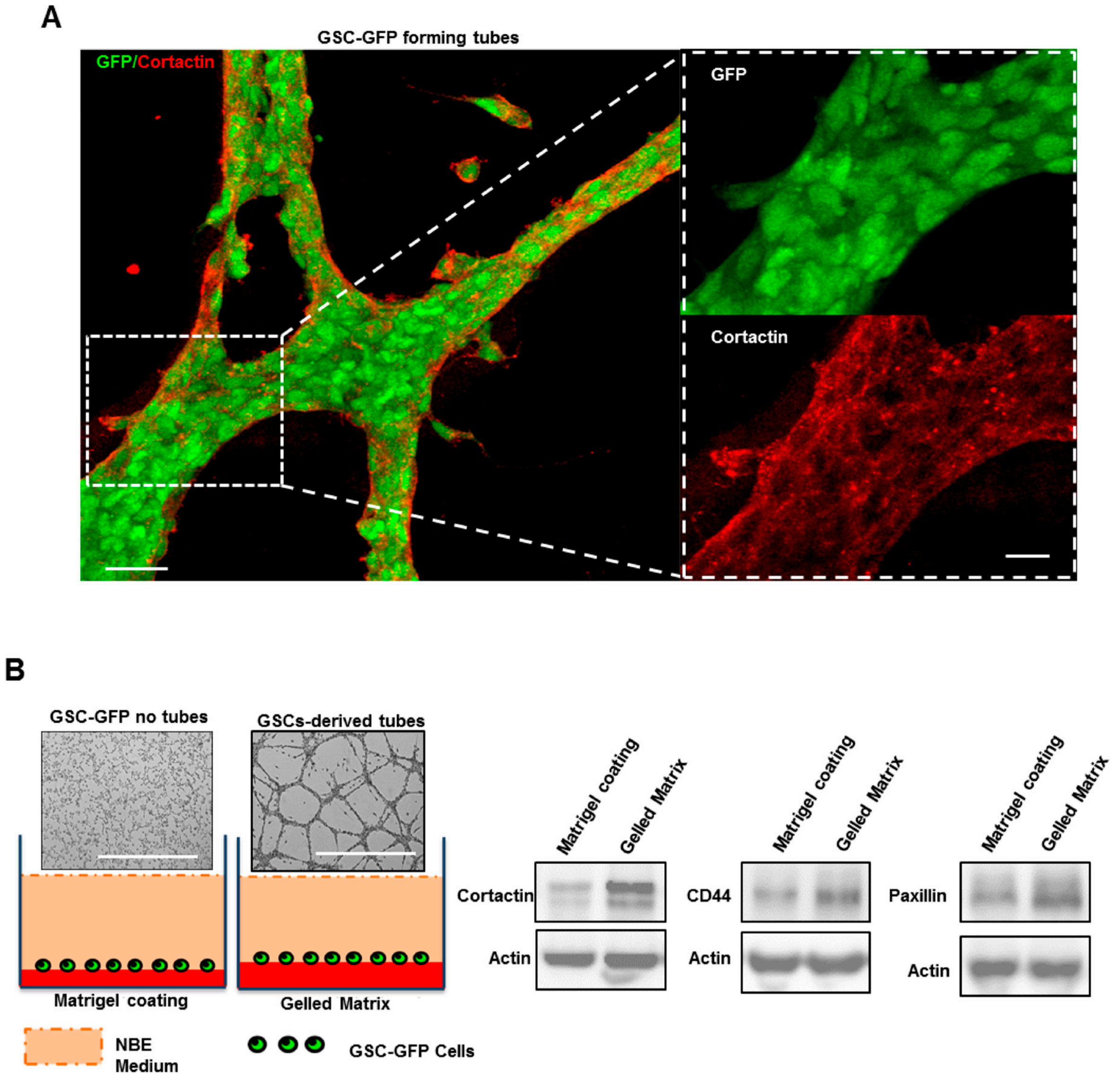

Supplementary Figure 4: Invadopodia formation is highly required during tubes assembly. (**A**) Confocal images of GSC-GFP-derived tubes fixed and stained for the invadopodia marker cortactin. Examination of magnified images (boxed region) revealed zones where cortactin accumulated at dot-like invadopodium-puncta. (**B**) Cell lysates of GSCs seeded on matrigel-coated or gelled matrix substrates were analyzed by Western blotting and probed for cortactin, CD44, paxillin and actin as a loading control. Tube formation over the gelled matrix was accompanied by an increase in the protein level of invadopodia components. Bars: (A) 50 µm; 20 µm; (B) 100 µm.

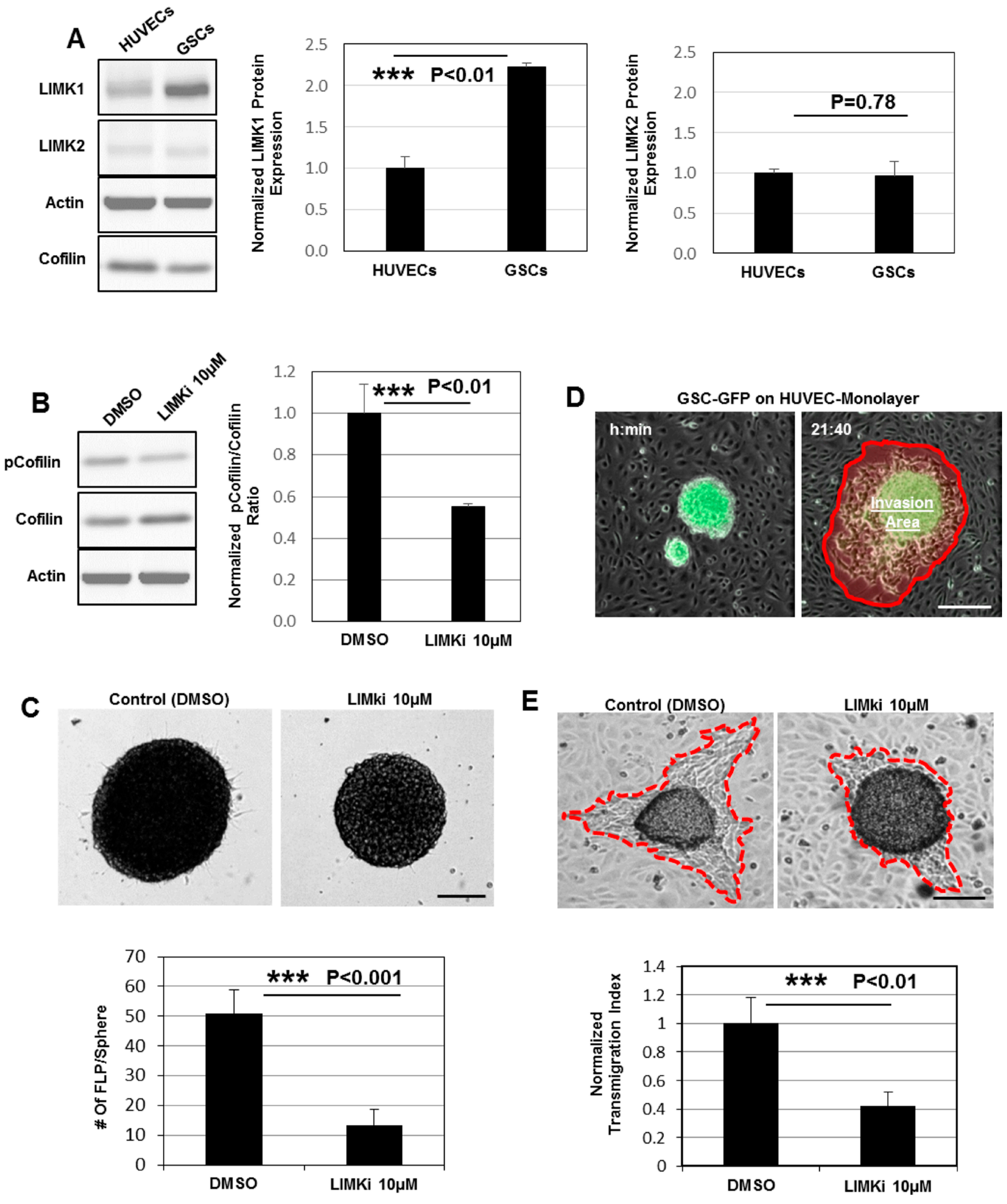

Supplementary Figure 5: ***In vitro*** **effects of LIM kinases inhibitor (LIMKi) on GSCs invasive migration and tumor cells characteristics.** (**A**) Cell lysates of GSC and HUVEC cells were analyzed by Western blotting and probed for LIMK1, LIMK2, cofilin, and actin as a loading control. Protein quantification revealed that the endogenous level of LIMK1 was at least 100% higher in GSCs, as compared to HUVEC cells; $^{***}P < 0.01$. In contrast, no difference was observed in LIMK2 and cofilin protein levels between GSC and HUVEC cells ($n = 3$). (**B**) Lysates of GSCs treated with DMSO (control condition) or 10 µM LIMKi were plotted for p-cofilin, cofilin and actin as a loading control. By quantifying the pcofilin/cofilin ratio we observed that LIMKi-treated cells exhibited at least 45% decrease on cofilin phosphorylation; $^{***}P < 0.01$ ($n = 4$). (**C**) LIMKi effects on cells migration tendency as measured by the number of FLP formed per sphere; $^{***}P < 0.001$ ($n = 3$). (**D**) Representative images extracted from a time series of GSC-derived spheroids seeded on top of HUVEC-based confluent monolayer. GSCs progressive unloading, disrupted HUVEC-based layer and created over time an invasion area (zone enclosed within the red line). (**E**) LIMKi effects on GSCs transmigration capacities on HUVEC-derived confluent monolayers; $^{***}P < 0.01$ ($n = 3$). All graphs are presented as means ± SD; a.u; arbitrary unit. Differences with a probability level $P < 0.05$ were considered significant in one-way ANOVA. Bars: (C) 50 µm; (D) 50 µm; (E) 50 µm.

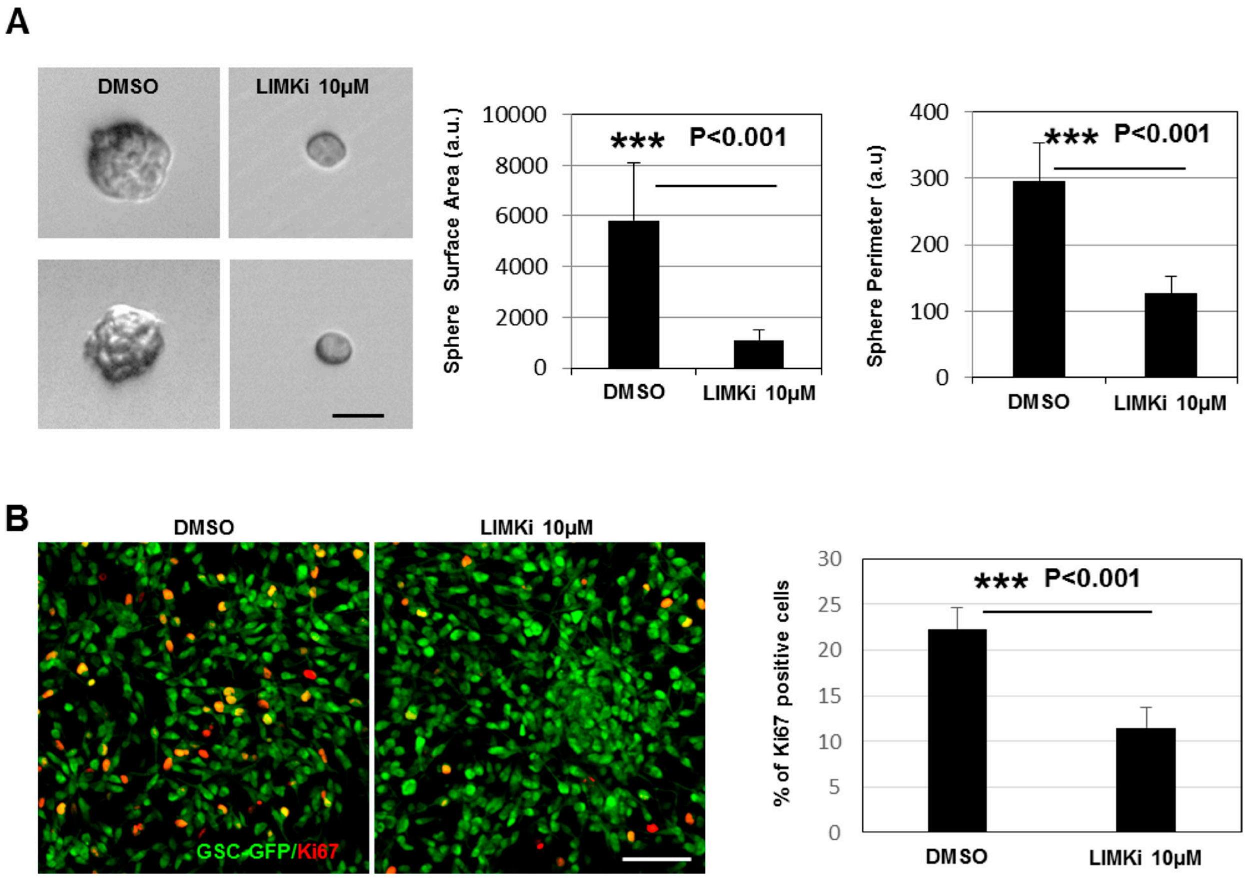

Supplementary Figure 6: *In vitro* effects of LIM kinases inhibitor (LIMKi) on GSCs growth. (**A**) Representative micrographs of spheres generated by GSC cells seeded at a low density in the presence or not of 10 µM LIMKi. LIMKi addition dramatically impacted on spheres size as indicated by spheres surface area and perimeter; $^{***}P < 0.001$ ($n = 3$). (**B**) Representative images of Ki67 staining of DMSO- or LIMKi-treated GSC-GFP cells after 48 h of treatment. Quantification of Ki67-positive cells revealed that LIMKi treatment significantly impacted on GSCs proliferation; $^{***}P < 0.001$ ($n = 3$). All graphs are presented as means ± SD; a.u; arbitrary unit. Differences with a probability level $P < 0.05$ were considered significant in one-way ANOVA. Bars: (A) 20 µm; (B) 50 µm.

Supplementary Video 1: Phase contrast and live fluorescent imaging of GSC-GFP cells forming tubes on gelled-matrix substrates. Frames were collected every 5 min for 23.5 h. Images are displayed at 15 frames/sec. See Supplementary_Video_1

Supplementary Video 2: Phase contrast and live fluorescent imaging of GFP GSC-derived spheroids seeded on top of HUVEC-based confluent monolayer. Frames were collected every 5 min for ~21.5 h. Images are displayed at 15 frames/sec. See Supplementary_Video_2
